# The electrophysiological effects of cannabidiol on action potentials and transmembrane potassium currents in rabbit and dog cardiac ventricular preparations

**DOI:** 10.1007/s00204-021-03086-0

**Published:** 2021-05-24

**Authors:** Leila Topal, Muhammad Naveed, Péter Orvos, Bence Pászti, János Prorok, Ákos Bajtel, Tivadar Kiss, Boglárka Csupor-Löffler, Dezső Csupor, István Baczkó, András Varró, László Virág, Norbert Jost

**Affiliations:** 1 Department of Pharmacology and Pharmacotherapy, Faculty of Medicine, University of Szeged, Dóm tér 12, 6720 Szeged, Hungary; 2 ELKH-SZTE Research Group for Cardiovascular Pharmacology, Eötvös Loránd Research Network, Szeged, Hungary; 3 Department of Pharmacognosy, Faculty of Pharmacy, University of Szeged, Szeged, Hungary; 4 Institute for Translational Medicine, Medical School, University of Pécs, Pécs, Hungary; 5 Department of Pharmacology and Pharmacotherapy, Interdisciplinary Excellence Centre, University of Szeged, 6720 Szeged, Hungary

**Keywords:** Cannabidiol, Electrophysiology, Action potential, Potassium currents, Rabbit, Dog

## Abstract

Cannabis use is associated with known cardiovascular side effects such as cardiac arrhythmias or even sudden cardiac death. The mechanisms behind these adverse effects are unknown. The aim of the present work was to study the cellular cardiac electrophysiological effects of cannabidiol (CBD) on action potentials and several transmembrane potassium currents, such as the rapid (I_Kr_) and slow (I_Ks_) delayed rectifier, the transient outward (I_to_) and inward rectifier (I_K1_) potassium currents in rabbit and dog cardiac preparations. CBD increased action potential duration (APD) significantly in both rabbit (from 211.7 ± 11.2. to 224.6 ± 11.4 ms, *n* = 8) and dog (from 215.2 ± 9.0 to 231.7 ± 4.7 ms, *n* = 6) ventricular papillary muscle at 5 µM concentration. CBD decreased I_Kr_, I_Ks_ and I_to_ (only in dog) significantly with corresponding estimated EC_50_ values of 4.9, 3.1 and 5 µM, respectively, without changing I_K1_. Although the EC_50_ value of CBD was found to be higher than literary C_max_ values after CBD smoking and oral intake, our results raise the possibility that potassium channel inhibition by lengthening cardiac repolarization might have a role in the possible proarrhythmic side effects of cannabinoids in situations where CBD metabolism and/or the repolarization reserve is impaired.

## Introduction

Cannabis has been one of the most abused hallucinogenic drugs since ancient times with an estimated 150 million consumers worldwide (Kalla et al. 2018). Moreover, the increasingly widespread use of e-cigarettes, the number of people inhaling cannabinoids might even be higher. In addition, the use of cannabis products for medicinal purposes is increasing globally. The enhanced general interest for the use of cannabis and cannabis-derived products was facilitated following the discovery of the cannabinoid system in humans (Sierra et al. 2018). The subsequent new findings on biological actions of cannabinoids on the central nervous system and immune functions attracted further attention. At present, there are cannabis-based drugs on the market with well-defined indications, including treatment of nausea and vomiting following chemotherapy, anorexia, pain related to cancer, spasticity and pain associated with multiple sclerosis, and Dravet and Lennox-Gastaut syndromes (Fraguas-Sánchez and Torres-Suárez 2018). These drugs contain known amounts of CBD and/or THC in pure form or as herbal extract (Fraguas-Sánchez and Torres-Suárez 2018). In addition to the use of CBD-containing products, CBD oil is very common with several, clinically unsupported indications. The consumption of cannabinoids, particularly CBD, which is enriched in numerous products, can be higher in case of the intake of CBD oils than in case of smoking cannabis. The consumption of cannabinoids, particularly CBD, which is enriched in numerous products, can be higher in case of the intake of CBD oils than in case of smoking cannabis. At high temperature, the majority of CBD is broken down (Czégény et al. 2021), whilst from CBD oils (in fact CBD dissolved in vegetable oils) containing up to 20% CBD, a significant amount of CBD is absorbed.

The possible cardiovascular side effects of cannabinoid use have been indicated in several reports, ranging from arrhythmias to myocardial infarction and even sudden cardiac death (Pacher et al. 2018). According to a cohort study, marijuana smokers can have a 4.8-fold increase of risk developing acute myocardial infarction following the first hour of cannabinoid exposure (Mittleman et al. 2001). On the other hand, other reports do not support the link between cannabis use and cardiovascular events (Singh et al. 2019). Accordingly, an important comprehensive study assessed data for 316,397 cannabis users and 20,499,215 non-users found that cannabis use was an independent predictor of heart failure (Kalla et al. 2018). Although the mechanisms explaining these observations are poorly understood, the effects of cannabinoids exerted via the G protein-coupled cannabinoid receptors are suspected to play key roles. In addition, numerous studies reported proarrhythmic properties of cannabinoids including ventricular arrhythmias and even sudden cardiac death (Courts et al. 2016; Ozturk et al. 2019; Manolis et al. 2019). However, the mechanism of these arrhythmias remains unclear (Ozturk et al. 2019). It was reported earlier and also recently that certain voltage-gated ion channels like cardiac sodium, calcium (Al Kury et al. 2014), hERG and Kv4.3 channels (Amoros et al. 2010) might be also related to the reported cardiac effects of cannabinoids, but the possible effects of CBD on various cardiac potassium currents which play a crucial role in cardiac repolarization have not been studied yet in detail. Such transmembrane ion currents in cardiac ventricular muscle are the rapid (I_Kr_) and slow (I_Ks_) delayed rectifier potassium currents, the transient outward (I_to_) and inward rectifier (I_K1_) potassium currents, all important for cardiac repolarization. Several cardiac and non-cardiac drugs are known to inhibit I_Kr_ (also called hERG ion channel) and consequently they prolong cardiac QT interval and enhance dispersion of repolarization. The latter has been associated with the development of life-threatening arrhythmias. Therefore, the official drug development procedure requires an early screening of whether a potential drug candidate has any activities on the hERG channels (Sanguinetti and Tristani-Firouzi 2006). However, drug effects on cardiac repolarization cannot be accurately estimated by measuring hERG channel and currents (Orvos et al. 2019), since drugs can also affect cardiac repolarization and action potential by acting on different currents other than hERG or I_Kr_.

Therefore, in the present study the aim was to investigate the effect of CBD, a major cannabinoid, on cardiac ventricular action potential and on several cardiac transmembrane currents to provide further experimental data for the elucidation of the possible mechanisms of its adverse cardiac electrophysiological effects.

## Methods

### Animals and materials

All experiments were carried out in compliance with the Guide for the Care and Use of Laboratory Animals (USA NIH publication NO 85-23, revised 1996) and conformed to the Directive 2010/63/EU of the European Parliament. The protocols have been approved by the Ethical Committee for the Protection of Animals in Research of the University of Szeged, Szeged, Hungary (approval numbers: I-74-15-2017 and I-74-24-2017) and by the Department of Animal Health and Food Control of the Ministry of Agriculture and Rural Development (authority approval numbers XIII/3330/2017 and XIII/3331/2017).

### Conventional microelectrode technique

Action potentials were recorded in right ventricular trabecular or papillary muscle preparations obtained from dog or rabbit hearts using conventional microelectrode techniques as described earlier in detail (Jost et al. 2013; Orvos et al. 2019).

Preparations were individually mounted in a tissue chamber with a volume of 50 ml. During experiments modified Locke’s solution was used, containing (in mM): NaCl 128.3, KCl 4, CaCl_2_ 1.8, MgCl_2_ 0.42, NaHCO_3_ 21.4 and glucose 10. The pH of this solution was set between 7.35 and 7.4 when gassed with the mixture of 95% O_2_ and 5% CO_2_ at 37 °C. Each preparation was stimulated through a pair of platinum electrodes in contact with the preparation using rectangular current pulses of 1–3 ms duration at twice of the threshold strength at a constant basic cycle length of 1000 ms for ventricular preparations. These stimuli were delivered for at least 60 min allowing the preparation to equilibrate before the measurements were initiated. Transmembrane potentials were recorded using conventional glass microelectrodes, filled with 3 M KCl and having tip resistances of 5–20 MΩ, connected to the input of a high impedance electrometer (Experimetria, type 309, Budapest, Hungary) which was coupled to a dual beam oscilloscope.

The resting potential (RP), action potential amplitude (APA), maximum upstroke velocity (V_max_) and APD measured at 50% and 90% of repolarization (APD_50_ and APD_90_, respectively) were determined off-line using an in-house developed software (APES) running on a computer equipped with an ADA 3300 analogue-to-digital data acquisition board (Real Time Devices, Inc., State College, Pennsylvania) having a maximum sampling frequency of 40 kHz.

Attempts were made to maintain the same impalement throughout each experiment. In case an impalement became dislodged, adjustment was attempted, and if the action potential characteristics of the re-established impalement deviated by less than 5% from the previous measurement, the experiment continued.

### Voltage-clamp measurements

Ventricular myocytes were enzymatically dissociated from canine or rabbit hearts as described earlier in detail (Jost et al. 2013; Orvos et al. 2019). One drop of cell suspension was placed in a transparent recording chamber mounted on the stage of an inverted microscope (Olympus IX51, Olympus, Tokyo, Japan), and individual myocytes were allowed to settle and adhere to the chamber bottom for at least 5–10 min before superfusion was initiated and maintained by gravity. Only rod-shaped cells with clear striations were used. HEPES-buffered Tyrode’s solution (composition in mM: NaCl 144, NaH_2_PO_4_ 0.4, KCl 4.0, CaCl_2_ 1.8, MgSO_4_ 0.53, glucose 5.5 and HEPES 5.0, at pH of 7.4) served as the normal superfusate.

Micropipettes were fabricated from borosilicate glass capillaries (Science Products GmbH, Hofheim, Germany), using a P-97 Flaming/Brown micropipette puller (Sutter Co, Novato, CA, USA), and had a resistance of 1.5–2.5 MOhm when filled with pipette solution. The membrane currents were recorded with Axopatch-200B amplifiers (Molecular Devices, Sunnyvale, CA, USA) by means of the whole-cell configuration of the patch-clamp technique. The membrane currents were digitized with 250 kHz analogue-to-digital converters (Digidata 1440A, Molecular Devices, Sunnyvale, CA, USA) under software control (pClamp 10, Molecular Devices, Sunnyvale, CA, USA). Experiments were carried out at 37 °C.

#### Measurement of potassium currents

The inward rectifier (I_K1_), transient outward (I_to_), rapid (I_Kr_) and slow (I_Ks_) delayed rectifier potassium currents were recorded in HEPES-buffered Tyrode’s solution. The composition of the pipette solution (in mM) was the following: KOH 110, KCl 40, K_2_ATP 5, MgCl_2_ 5, EGTA 5 and HEPES 10 (pH was adjusted to 7.2 by aspartic acid). 1 µM nisoldipine was added to the bath solution to block I_CaL_. When I_Kr_ was recorded I_Ks_ was inhibited using the selective I_Ks_ blocker HMR 1556 (0.5 µM). During I_Ks_ measurements (a transmembrane current strongly depending from cAMP and protein kinase A, PKA; Christian et al. 2011), I_Kr_ was blocked by 0.5 µM dofetilide and the bath solution contained 0.1 µM forskolin.

### Data analysis

All data are expressed as means ± SEM. The “*n*” number refers to the number of experiments (*i.e.* the number of cells in case of patch-clamp and the number of ventricular muscle preparations—papillary or trabecular muscle—in case of action potential measurements). Statistical analysis was performed with Student's *t* test for paired data. The results were considered statistically significant when *P* was < 0.05.

## Results

**Fig. 1:**
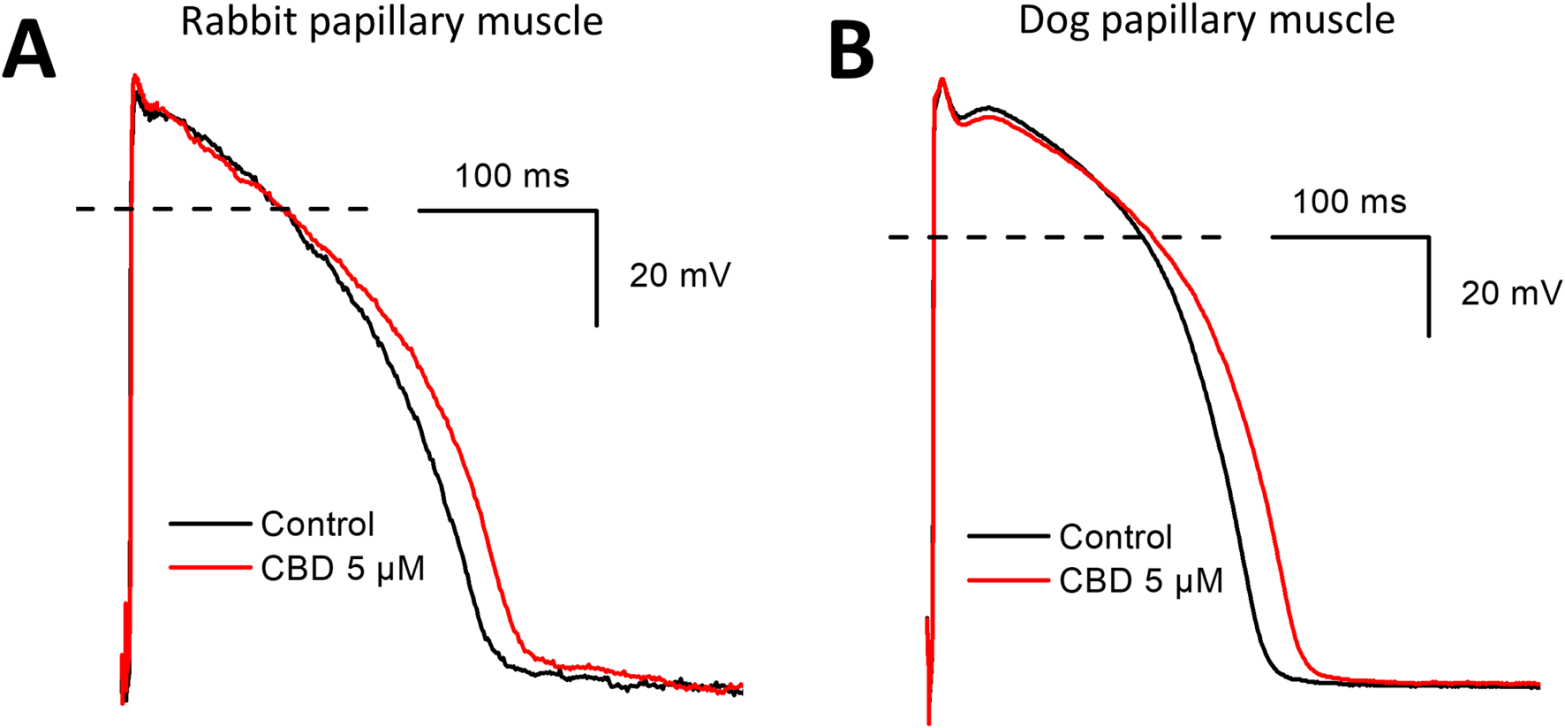
Effect of CBD on the action potentials recorded from rabbit (panel **A**) and dog (panel **B**) papillary muscles. Dashed lines indicate zero mV levels

The cardiac cellular electrophysiological effect of CBD was studied on various transmembrane ionic currents by the whole-cell configuration of the patch-clamp technique in native rabbit and dog ventricular myocytes and on action potentials in rabbit and dog ventricular papillary muscles by the conventional microelectrode technique. Figure 1 and Table 1 show that CBD lengthens action potential duration (APD_90_) significantly at the concentration of 5 µM without changing other action potential parameters significantly.

**Table 1:** Effect of acute exposure to CBD on the action potential parameters in rabbit and dog right ventricular papillary muscle preparations

Parameters	Rabbit ventricular muscle (*n* = 8)		Dog ventricular muscle (*n* = 6)	
	Control	CBD 5 µM	Control	CBD 5 µM
RP (mV)	− 84.1 ± 2.2	− 82.7 ± 1.7	− 84.7 ± 1.7	− 84.7 ± 2.3
APA (mV)	105.2 ± 3.0	106.4 ± 3.0	118.4 ± 3.3	120.4 ± 2.2
V_max_ (V/s)	120.3 ± 20.6	113.0 ± 17.1	186.4 ± 21.7	201.0 ± 25.2
APD_50_ (ms)	171.8 ± 13.6	**183.0 ± 12.8**	178.3 ± 8.2	193.1 ± 4.5
APD_90_ (ms)	211.7 ± 11.2	**224.6 ± 11.4**	215.2 ± 9.0	**231.7 ± 4.7**

Bold values are considered to be statistically significant (*P* < 0.05 versus control)

*P* < 0.05 versus control

*RP* resting membrane potential, *APA* action potential amplitude, *V*_*max*_ maximum upstroke velocity

APD_50_ and APD_90_ action potential duration measured at 50 and 90% of repolarization

**Fig. 2:**
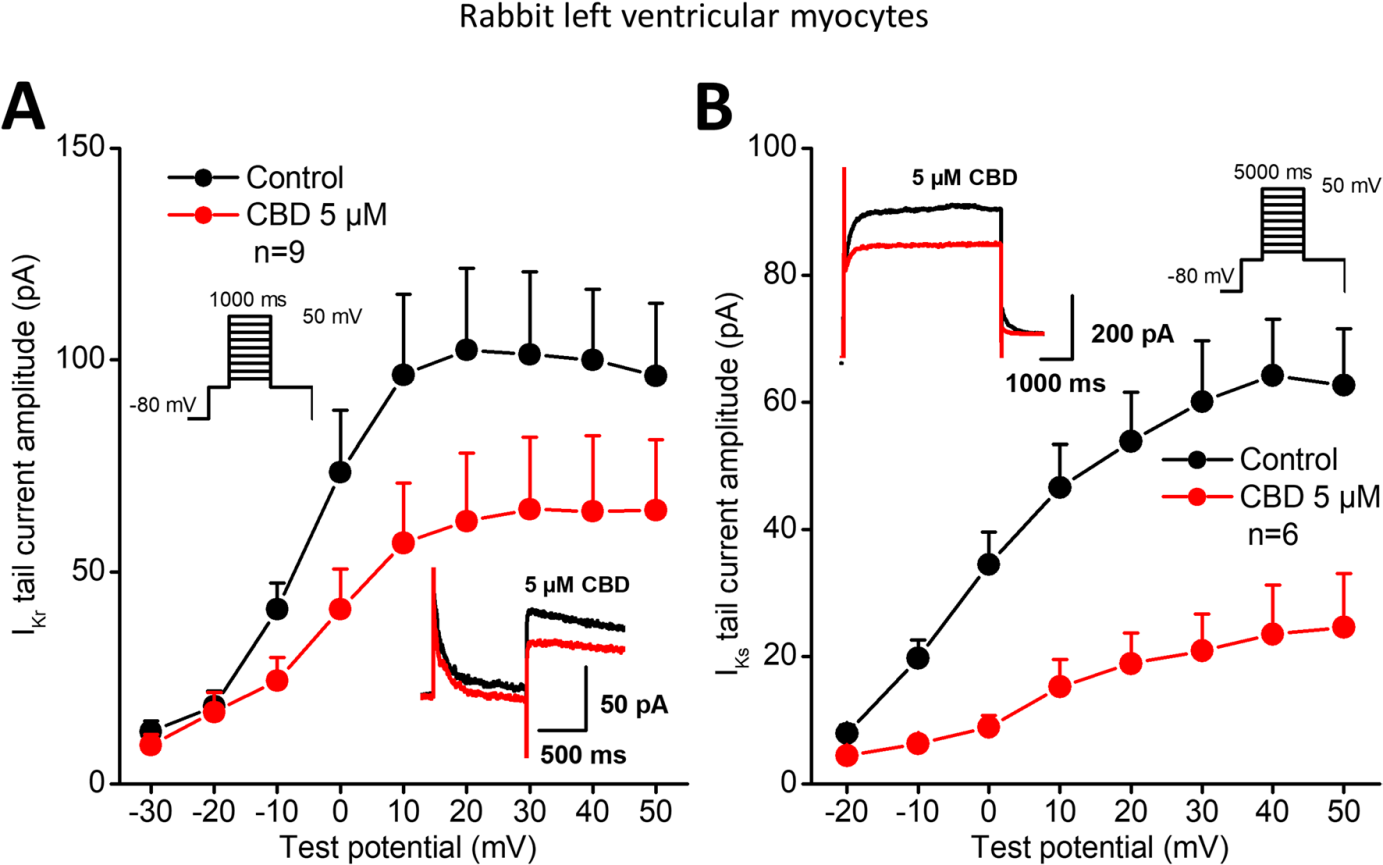
Effect of CBD on the rapid (I_Kr_) and slow (I_Ks_) delayed rectifier potassium currents. Panels show current–voltage curves for I_Kr_ (panel **A**) and for I_Ks_ (panel **B**) in control conditions and after application of 5 µM CBD. Insets indicate the voltage protocols and original I_Kr_ and I_KS_ current records in control and in the presence of CBD. Data are expressed as means ± SEM

**Fig. 3:**
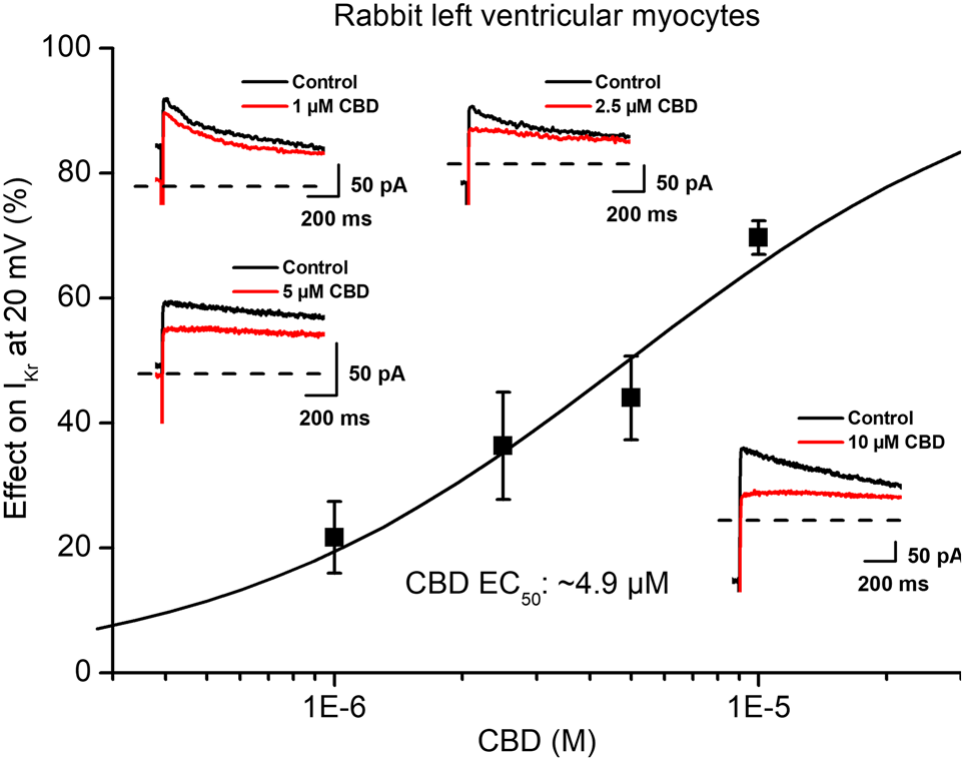
Effect of CBD on the rapid (I_Kr_) delayed rectifier potassium currents. The panel displays CBD concentration–response curve indicating an estimated EC_50_ value of 4.9 µM for I_Kr_ blockade. The insets show the tail current section of original I_Kr_ current traces in control conditions and in the presence of 1 µM, 2.5 µM, 5 µM and 10 µM CBD recorded from rabbit left ventricular myocytes after a 1 s long pulse to 20 mV test potential with pulsing cycle length of 20 s. I_Kr_ deactivating tail current was measured at -40 mV. The dashed lines refer to the baseline for I_Kr_ tail current level after the test pulse at − 40 mV. Data are expressed as means ± SEM

Whole-cell patch-clamp experiments in rabbit cardiac ventricular myocytes revealed significant inhibition of the rapid delayed rectifier potassium current (I_Kr_) (Figs. 2A and 3) with an estimated EC_50_ value of 4.9 µM. I_Kr_ was activated by 1000 ms long depolarizing voltage pulses with pulse frequency of 0.05 Hz to the potentials ranging from − 30 mV to 50 mV and then the cell was repolarized to − 40 mV. The deactivating tail current at − 40 mV after the test pulse was assessed as I_Kr_. The holding potential was − 80 mV.

**Fig. 4:**
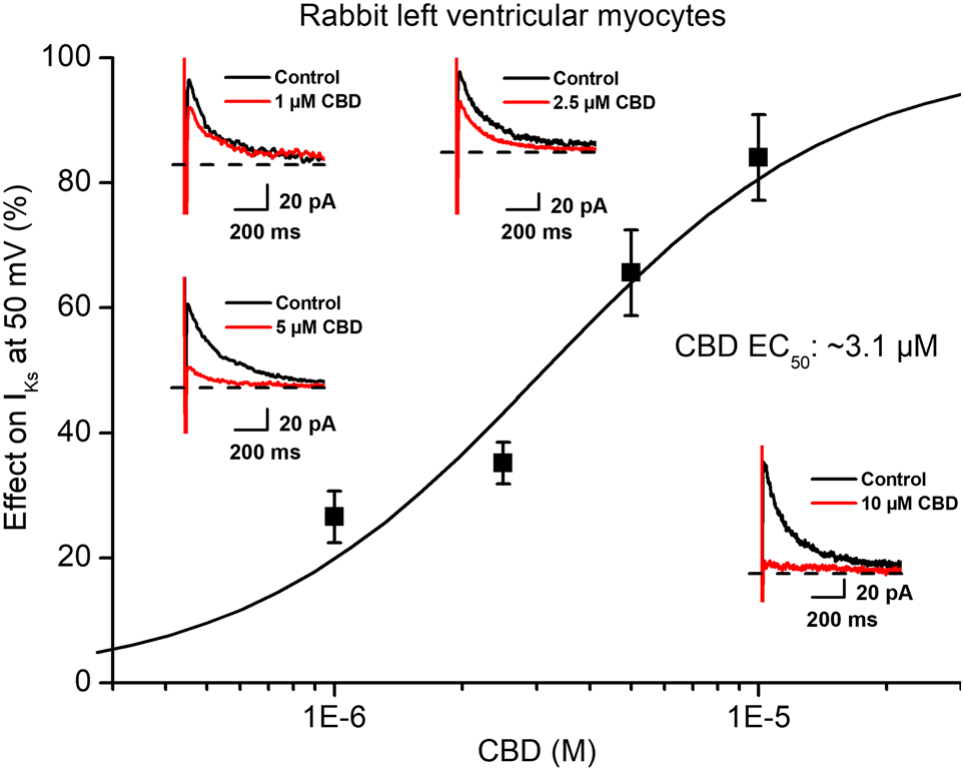
Effect of CBD on the slow (I_Ks_) delayed rectifier potassium currents. The panel displays CBD concentration–response curve indicating an estimated EC_50_ value of 3.1 µM for I_Ks_ blockade. The insets show the tail current section of original I_Ks_ current traces in control conditions and in the presence of 1 µM, 2.5 µM, 5 µM and 10 µM CBD recorded from rabbit left ventricular myocytes after a 5 s long pulse to 50 mV test potential with pulsing cycle length of 10 s. I_Ks_ deactivating tail current was measured at -40 mV. The dashed lines refer to the baseline for I_Ks_ tail current level after the test pulse at − 40 mV. Data are expressed as means ± SEM

In similar experiments in rabbit myocytes CBD depressed the slow delayed rectifier potassium current (I_Ks_, Fig. 2B) with an estimated EC_50_ value of 3.1 µM (Fig. 4), after 20 mV 5 s long test pulse measured at − 40 mV. I_Ks_ was recorded similarly to I_Kr_. After 5 s long depolarizing voltage pulses to various test potentials with pulse frequency of 0.1 Hz the cell was repolarized to − 40 mV and the tail current amplitude was measured.

**Fig. 5:**
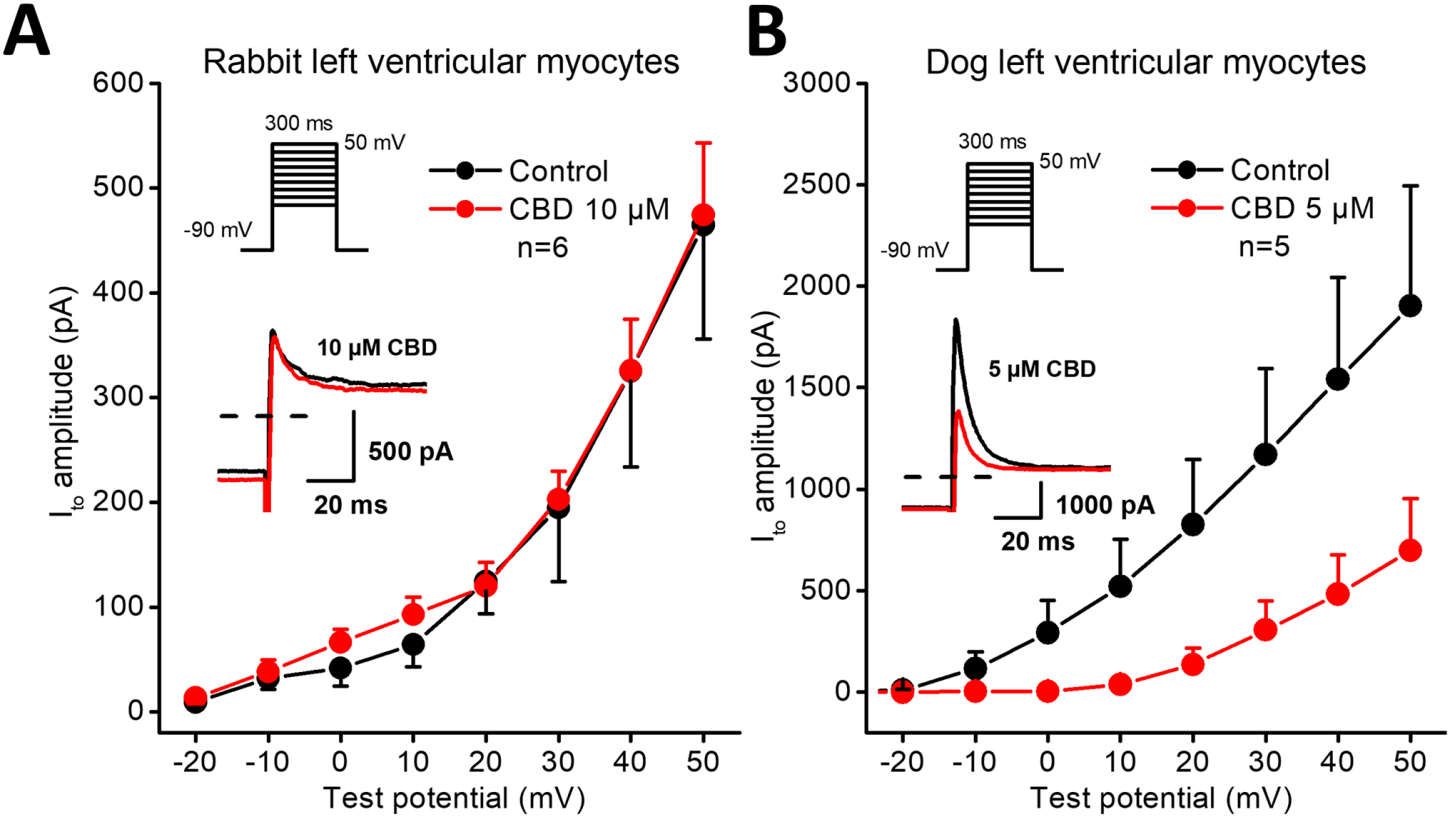
Effect of CBD on the transient outward potassium current (I_to_) in rabbit and dog ventricular myocytes. Panels show current–voltage curves for I_to_ in control conditions and after application of CBD in rabbit (panel **A**) and in dog (panel **B**) ventricular myocytes. Insets indicate the voltage protocols and original I_to_ current records in control and in the presence of CBD. Dashed lines indicate zero current levels. Data are expressed as means ± SEM

**Fig. 6:**
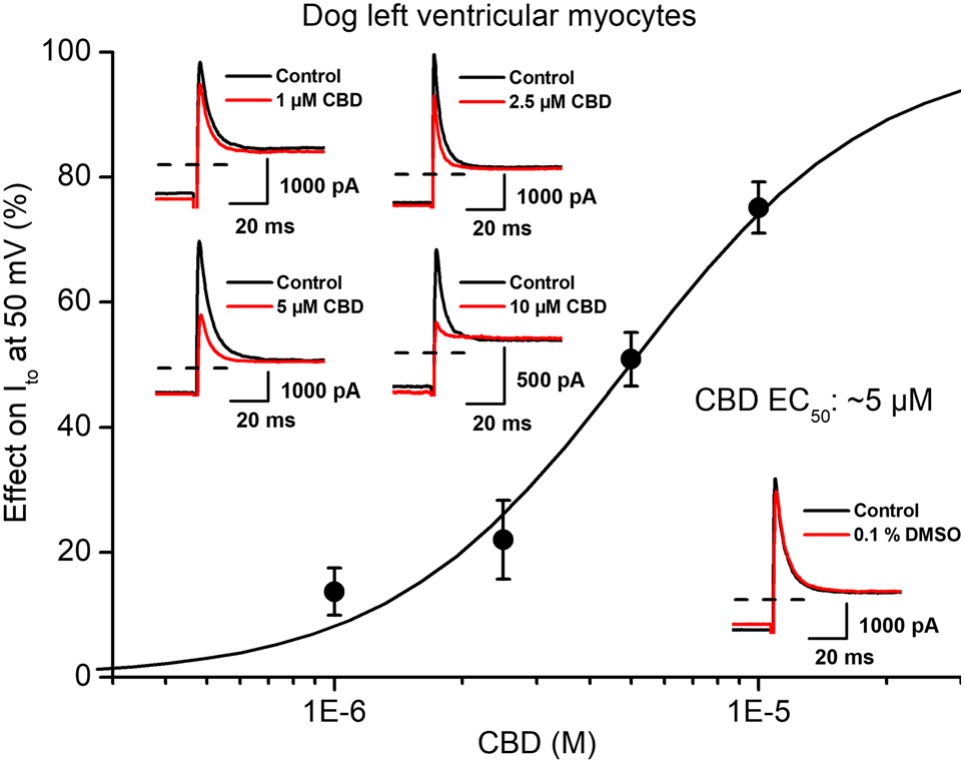
Effect of CBD on the transient outward potassium current (I_to_) in dog ventricular myocytes. The panel displays CBD concentration–response curve indicating an estimated EC_50_ value of 5 µM for I_to_ blockade. Insets show original I_to_ current traces in control conditions and in the presence of 1 µM, 2.5 µM, 5 µM and 10 µM CBD recorded from dog left ventricular myocytes after a 300 ms long pulse to 50 mV test potential with pulsing cycle length of 3 s. The inset on right-bottom displays original I_to_ current traces in control conditions and in the presence of the solvent (0.1% DMSO). Dashed lines indicate zero current levels. Data are expressed as means ± SEM

CBD even in the high concentration of 10 µM concentration did not influence the transient outward potassium current (I_to_) in rabbit (Fig. 5A) but decreased it significantly in dog (Fig. 5B) ventricular myocytes with an estimated EC_50_ value of 5 µM (Fig. 6). I_to_ was activated by 300 ms long depolarizing voltage pulses arising from the holding potential of − 90 mV to test potentials gradually increasing up to 50 mV. The pulse frequency was 0.33 Hz.

**Fig. 7:**
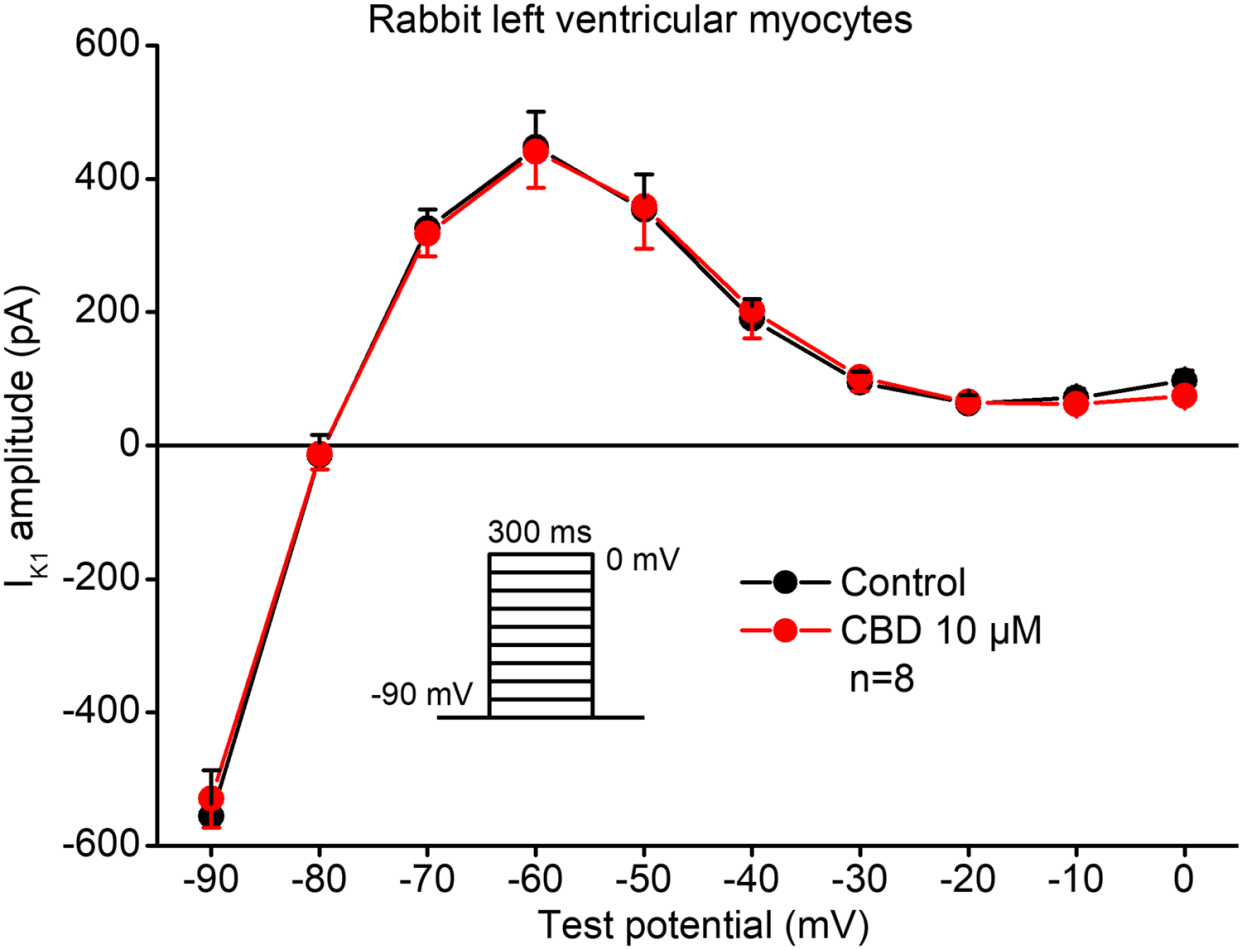
Lack of effect of CBD on the inward rectifier potassium current in rabbit left ventricular myocytes. The panel shows steady-state current–voltage curves for I_K1_ in control conditions and after application of 10 µM CBD in rabbit left ventricular myocytes. Inset indicates the voltage protocol. Data are expressed as means ± SEM

As Fig. 7 indicates, CBD did not significantly change the inward rectifier potassium current (I_K1_) even at the high, 10 µM concentration. I_K1_ current was measured as the steady-state current level at the end of the 300 ms long voltage pulse in the voltage range of − 100 to 0 mV with a pulse frequency of 0.33 Hz. The holding potential was − 90 mV.

## Discussion

The main result of this study is that 5 µM CBD prolongs repolarization. This effect on repolarization in rabbit and dog papillary muscle can be best explained by the multiple effects CBD exerts on various potassium channels. Accordingly, as our previous results (Orvos et al. 2020) indicated, CBD administration at lower concentrations (1, 2.5 and 5 µM) resulted in hERG/I_Kr_ depression and a consequent lengthening of APD_90_, but this effect was counterbalanced by the inhibition of inward Ca^2+^ and Na^+^ currents following CBD application at the high concentration of 10 µM. Similar effects were reported earlier with quinidine, an antiarrhythmic drug, with established proarrhythmic properties (Roden and Hoffman 1985; Varro et al. 1985).

A few previous studies with cannabinoids showed effects on various transmembrane ion channels such as inward sodium, (Al Kury et al. 2014; Ghovanloo et al. 2018; Orvos et al. 2020) inward calcium (Al Kury et al. 2014; Orvos et al. 2020), outward transient current (Li et al. 2012) and human Kv1.5 and Kv4.3 channels (Barana et al. 2010). In addition, in previous studies (Orvos et al. 2020) hERG/I_Kr_ channel inhibition and QT prolongation were also reported in anaesthetized rats (Yun et al. 2016) and guinea pig (Orvos et al. 2020) by a synthetic cannabinoid compound (JWH-030) and CBD. This synthetic cannabinoid compound structurally differs from CBDs and inhibited hERG channels with a relatively high EC_50_ (88.36 µM). Also, in rat ventricle hERG/I_Kr_ seems not as important for controlling repolarization as Kv4.2 and Kv1.5 channels. Therefore, the cannabinoid-evoked QT changes in rat most likely can be attributed to Kv1.5 and Kv4.2 rather than hERG channel inhibition. The finding of the present study that CBD inhibits I_to_ in dog but not in rabbit ventricular myocytes are in good agreement with the previously mentioned rat study, since in dog I_to_ is conducted Kv 4.3 (Han et al. 2002) but in rabbit by Kv 1.4 channels (Wang et al. 1999). Since the APD measurements in the present study were taken in subendocardial preparations, the latter effect on I_to_ may result in more pronounced repolarization dispersion in dog and human ventricle where in midmyocardial cells I_to_ is greater than in the subendocardium (Zicha et al. 2004).

According to human pharmacokinetic data, the C_max_ values for CBD can reach 0.35 µM and 0.58 µM during CBD smoking (19.2 mg) or following oral administration (400 mg), respectively (Millar et al. 2018). In the present experiments, CBD had inhibitory potency on both the hERG channel and I_Kr_ activity, with an EC_50_ value higher than literary C_max_ values in patients. This suggests small or negligible proarrhythmic risk in physiological conditions in healthy individuals. This is indeed in good agreement with clinical reports showing no significant QT_c_ prolongation in patients after CBD administration (Sellers et al. 2013). Also, in another clinical study, it was found that long term Sativex (THC + CBD) treatment evoked T wave changes in only 1 out of 146 patients (Serpell et al. 2013). Therefore, it is likely that in case of inhalation or oral use of cannabis-derived products, CBD itself may not represent a significant proarrhythmic risk. Based on the comparison of hERG or I_Kr_ activity, cardiac action potential duration, and QT prolongation against QT effects and reports of arrhythmogenic (torsade de pointes) potential of 100 drugs, a margin of 30-fold between hERG EC_50_ and C_max_ was proposed to be an acceptable degree of safety regarding arrhythmogenesis (Redfern et al. 2003). Taking into account the EC_50_ values for I_Kr_, I_Ks_ and I_to_ inhibition in our experiments (4.9, 3.1 and 5 µM, respectively), the ratios of EC_50_ and C_max_ values are in the range of about 8–9, which refers to moderately increased risk of arrhythmia. However, in patients who have considerably slower drug elimination due to certain concomitant diseases or in case of concurrent use of other drugs that inhibit the metabolism of CBD, higher C_max_ values can develop (Iffland and Grotenhermen 2017), and this may further increase the risk for arrhythmia development.

Moreover, when CBD intake is combined with pharmacological agents affecting cardiac repolarization, as well as in certain pathophysiological situations such as hypokalemia, or diseases like LQT syndrome, diabetes mellitus, HCM or heart failure where cardiac repolarization reserve (Varró and Baczkó 2011) or drug metabolism is impaired, CBD can have an additive effect, further increasing the proarrhythmic risk and the possible incidence of sudden cardiac death. Such additive interactions were reported both in animal experimental (Lengyel et al. 2007) and clinical settings (Wisniowska et al. 2016). The cardiovascular effects of CBD may only partly be attributed to its effects on transmembrane ion channels, the cardiovascular safety of this compound may be influenced by its activities on other targets, and by the presence of myocardial ischemia (Ferdinandy et al. 2019) as well. Therefore, further studies are needed to assess the unwanted cardiovascular effects of CBD and other cannabinoids both in vivo and in vitro studies, with special focus on the benefit-risk assessment of products with different cannabinoid content.

## Data Availability

The data underlying this article will be shared on reasonable request to the corresponding author.
